# Pulsed electromagnetic fields promote osteogenesis and osseointegration of porous titanium implants in bone defect repair through a Wnt/β-catenin signaling-associated mechanism

**DOI:** 10.1038/srep32045

**Published:** 2016-08-24

**Authors:** Da Jing, Mingming Zhai, Shichao Tong, Fei Xu, Jing Cai, Guanghao Shen, Yan Wu, Xiaokang Li, Kangning Xie, Juan Liu, Qiaoling Xu, Erping Luo

**Affiliations:** 1 Department of Biomedical Engineering, Fourth Military Medical University, Xi’an, China; 2 Department of Radiation Oncology, PLA 302 Hospital, Beijing, China; 3 Department of Endocrinology, Xijing hospital, Fourth Military Medical University, Xi’an, China; 4 Institute of Orthopaedics, Xijing hospital, Fourth Military Medical University, Xi’an, China; 5 Department of Nursing, Fourth Military Medical University, Xi’an, China

## Abstract

Treatment of osseous defects remains a formidable clinical challenge. Porous titanium alloys (pTi) have been emerging as ideal endosseous implants due to the excellent biocompatibility and structural properties, whereas inadequate osseointegration poses risks for unreliable long-term implant stability. Substantial evidence indicates that pulsed electromagnetic fields (PEMF), as a safe noninvasive method, inhibit osteopenia/osteoporosis experimentally and clinically. We herein investigated the efficiency and potential mechanisms of PEMF on osteogenesis and osseointegration of pTi *in vitro* and *in vivo*. We demonstrate that PEMF enhanced cellular attachment and proliferation, and induced well-organized cytoskeleton for *in vitro* osteoblasts seeded in pTi. PEMF promoted gene expressions in Runx2, OSX, COL-1 and Wnt/β-catenin signaling. PEMF-stimulated group exhibited higher Runx2, Wnt1, Lrp6 and β-catenin protein expressions. *In vivo* results via μCT and histomorphometry show that 6-week and 12-week PEMF promoted osteogenesis, bone ingrowth and bone formation rate of pTi in rabbit femoral bone defect. PEMF promoted femoral gene expressions of Runx2, BMP2, OCN and Wnt/β-catenin signaling. Together, we demonstrate that PEMF improve osteogenesis and osseointegration of pTi by promoting skeletal anabolic activities through a Wnt/β-catenin signaling-associated mechanism. PEMF might become a promising biophysical modality for enhancing the repair efficiency and quality of pTi in bone defect.

Bone defects resulting from trauma, non-union fractures or osteo-degenerative diseases are common and challenging clinical issues. Application of bone grafts to improve bone healing is a major therapy for bone defect, especially for the critically sized defect that the endogenous bone regeneration is inadequate to repair the damaged tissues^1^. Clinically, autograft and allograft are regarded to be the major grafting procedures for the surgeons due to their excellent osteoinductive and osteogenic properties^2^. However, significant limitations exist regarding the tissue availability and donor site morbidity for the autograft^3^. Moreover, the autograft has the risk of being absorbed at the implantation site. Allograft also possesses some drawbacks, such as the immunological rejection and requirement of intense logistic efforts due to limited tissue availability^4^. Thus, it is of great clinical significance to develop safe and economic alternative grafting materials for the repair of bone defect.

Titanium alloys have been extensively recognized as ideal endosseous implants because of their excellent mechanical properties, biocompatibility and corrosion resistance. However, the mismatch in the mechanical strength between metallic implants and surrounding natural bone can cause the stress-shielding effect and accelerate bone resorption, and thus increase the risk of implant loosening^5^. Recently developed titanium-based alloys with porous structure were able to effectively decrease the mismatch of elastic modulus between implants and bone tissues^6^,^7^. Moreover, the internal porosity forms interconnected pore channels for the transport of nutrient and metabolites^6^,^8^. However, it should be noted that titanium alloys, as bioinert materials, can be easily connected with bones in the form of mechanical interlock rather than chemical bonding^5^,^8^,^9^. Thus, titanium alloys even at porous structure are impossible to achieve adequate osseointegration as the nature bone, which is regarded as a major clinical limitation for not providing early fixation with reliable long-term stability as bone substitutes, especially for the osteoporotic patients^10^,^11^. Therefore, developing novel technique that can promote the bone ingrowth through the pores and speed up osseointegration processes of porous titanium alloys (pTi) holds great significance for increasing the efficiency and success rate of the repair of bone defect.

In the past four decades, substantial and growing evidence has shown that pulsed electromagnetic fields (PEMF) therapy as an alternative noninvasive method is capable of producing satisfying therapeutic effects on a wide range of bone diseases, such as fresh and nonunion fractures and osteoarthritis^12^,^13^,^14^. Several *in vivo* investigations have also demonstrated that PEMF stimulation could inhibit bone loss and improve bone quality in various osteoporotic animals^15^,^16^,^17^,^18^,^19^,^20^. The anti-osteoporotic efficiency of PEMF was further confirmed by several clinical investigations^21^,^22^. PEMF stimulation has been shown to promote proliferation and mineralization of osteoblasts *in vitro* and also inhibit osteoclastogenesis^23^,^24^,^25^,^26^. Investigations by our group and others have also demonstrated that PEMF stimulation was able to promote osteoblast functions *in vitro* and accelerate bone formation *in vivo* onside pure titanium surface^27^,^28^,^29^,^30^,^31^. However, we still lack critical knowledge regarding whether PEMF can promote the biocompatibility of bone cells with titanium implants with porous structure and accelerate osteogenesis and osseointegration of pTi in bone defect repair, which may have more significant clinical therapeutic significance. Moreover, the potential mechanisms by which PEMF regulate osteogenesis and osseointegration of pTi also remain poorly understood.

In the present study, the potential effects of PEMF stimulation on the biological performance of pTi were systematically evaluated both *in vitro* and *in vivo*. First, we investigated the impacts and underlying mechanisms of PEMF on *in vitro* osteoblast activities and functions in pTi. Then, the promotional effects of PEMF stimulation on the repair of bone defect by pTi implants were systematically evaluated via analyses for histological and histomorphometric parameters. Furthermore, the molecular signaling pathway mechanisms of PEMF on *in vivo* osteogenesis in pTi implants were also investigated.

## Materials and Methods

### Preparation of pTi implants

The porous Ti6Al4V implants with 70% porosity and 750 μm pore size were designed using the computer-aided design (CAD) software and fabricated using the electron beam melting system (EBM S12, Acram AB, Sweden) according to our previously described protocol^7^. All implants were sterilized with an autoclave at 121 °C with the pressure of 103 kPa for 2 h before use. The prepared pTi implant was scanned with micro-computed tomography (μCT, Y. Cheetah, YXLON, Germany) and scanning electron microscope (SEM, JSM-6460, JEOL, Japan) for visualizing its microstructure and morphology, respectively.

### PEMF stimulators

**Figure 1:**
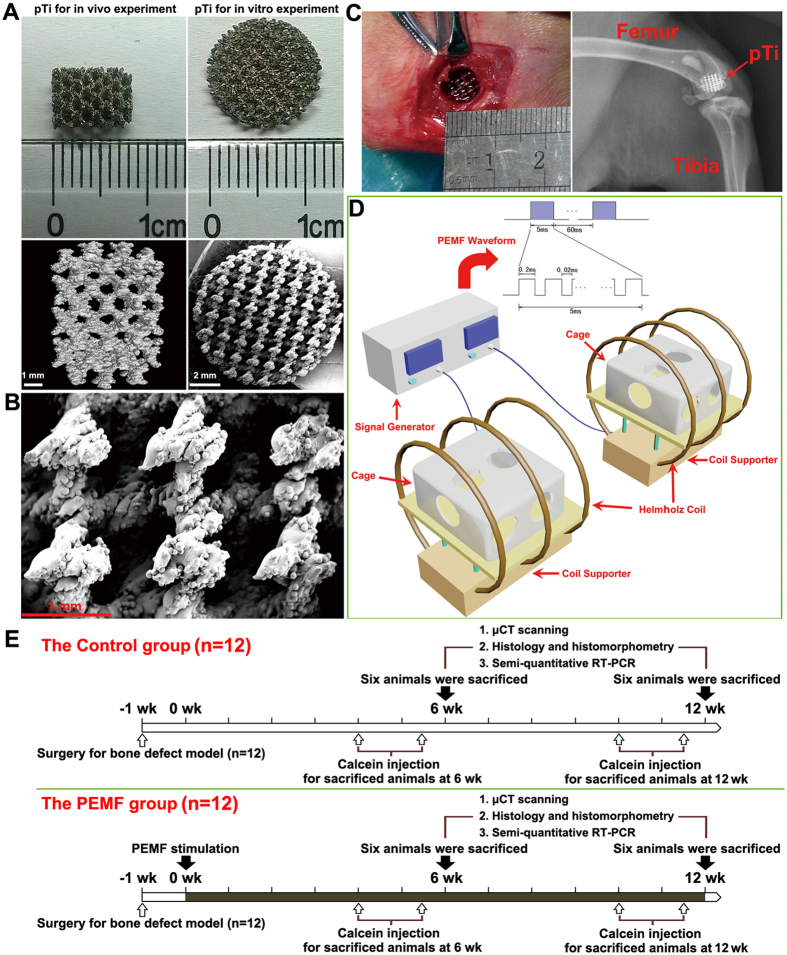
Characterization of pTi samples, PEMF system setups and *in vivo* experiment protocol. **(A)** Gross view and μCT scanning of pTi samples for *in vitro* and *in vivo* experiments. **(B)** Microstructural observation of pTi implants via SEM scanning. **(C)** Surgical photograph showing the cylindrical bone defect with 6.0 mm diameter and 8.0 mm length created in the femoral lateral condyle. A pTi implant was then transplanted into the bone defect sites and the accuracy of the defect location was further confirmed via X-ray scanning. **(D)** Schematic representation of the PEMF generator together with a Helmholtz coil assembly with three-coil array. The PEMF output waveform consisted of a pulsed burst (burst width, 5 ms; pulse width, 0.2 ms; pulse wait, 0.02 ms; burst wait, 60 ms; pulse rise, 0.3 μs; pulse fall, 2.0 μs) repeated at 15 Hz. The peak magnetic field within the Helmholtz coils was approximately 2.0 mT. **(E)** The experimental protocols for the present *in vivo* investigation.

As described in our previous studies^18^,^19^,^20^,^32^, the PEMF waveform used in the present *in vitro* or *in vivo* experiment consisted of a pulsed burst (burst width, 5 ms; pulse width, 0.2 ms; pulse wait, 0.02 ms; burst wait, 60 ms; pulse rise, 0.3 μs; pulse fall, 2.0 μs) repeated at 15 Hz (Fig. 1). This PEMF waveform has shown the efficiency on inhibiting bone loss induced by disuse, estrogen deficiency and type-1 diabetes in rats in our previous study^18^,^19^,^20^,^32^. The PEMF exposure system was composed of a signal generator and a Helmholtz coil assembly with three-coil array (Fig. 1). For the *in vivo* study, the three coils (80 cm diameter) were placed coaxially with 30.4 cm apart from each other, and the numbers of turns of the central coil and outside coils were 266 turns and 500 turns, respectively. The bottom of the plastic rabbit cage was aligned with the center of the coils to ensure that the rabbits were confined in the center of the electromagnetic fields. For the *in vitro* experiment, the interval distance between three coils (20 cm diameter) were 7.6 cm, and the turn numbers of the central coil and outside coils were 53 and 100, respectively. The assembly of three coils has been proved to exhibit significantly upgraded axial magnetic field uniformity and also show significantly decreased deviation of the magnetic field between the origin and any other off-axial point within the coils^19^,^33^. To calculate the current in the coils, a resistor of 2 Ω was placed in series with the coils and the voltage drop across the resistor was observed with an oscilloscope (Agilent Technologies, Santa Clara, CA, USA). The peak magnetic field of the coils was determined to be approximately 2.0 mT. The accuracy for the peak magnetic field measurement was further confirmed by using a Gaussmeter (Model 455 DSP, Lake Shore Cryotronics, Westerville, OH, USA). The measured background electromagnetic field was 50 ± 2 μT. In order to determine the induced electric field within the coils, a custom-designed electrical potential detecting circular coil (5 cm coil diameter, 1 mm coil diameter, 20 turns) was placed in the midcenter of the Helmholtz coils with the coil parallel to the Helmholtz coils. The current detecting coil was connected with the oscilloscope, and the induced peak electrical field was determined to be approximately 2 mV/cm.

### Cell culture

Osteoblast-like MC3T3-E1 cells (a cell line from C57BL/6 mouse calvaria) were obtained from American Type Culture Collection (ATCC, Manassas, VA, USA). Cells were maintained in α-Minimum Essential Medium (α-MEM, Hyclone, Logan, UT, USA) containing 10% fetal bovine serum (FBS, Hyclone) and 1% penicillin/streptomycin (Gibco, Carlsbad, CA, USA) in a water-saturated atmosphere of 5% CO_2_ at 37 °C. The α-MEM cell culture medium contained both ascorbic acid and sodium phosphate, which were important for MC3T3-E1 cells to maintain osteoblast-like phenotype. Cells were seeded into the pTi implants (12.0 mm diameter and 2.5 mm thickness) at a density of 5 × 10^4^ cells/ml for 12 h. Then, cells in the PEMF group were subjected to 2 h/day PEMF stimulation for 3 days. In the Control group, cells were placed within the inactivated Helmholtz coils to exhibit sham PEMF exposure.

### *In vitro* osteoblast attachment, morphology and proliferation

To evaluate the cell attachment and morphology, MC3T3-E1 cells after PEMF or sham exposure were fixed in 4% formaldehyde solution for 5 min and then permeabilized with 0.1% Triton X-100. Cells were stained with 50 mg/ml FITC (Sigma, St. Louis, MO, USA) for 40 min followed by 40,60-diamidino-2-phenylindole (DAPI, Beyotime Institute of Biotechnology, Jiangsu, China) staining for 5 min. Cells were then imaged and analyzed under a confocal microscope (FV1000, Olympus, Tokyo, Japan) in five randomly selected fields of view. For determining the cell proliferation, 3-(4,5-dimethylthiazol-2-yl)-2,5-diphenyltetrazolium bromide (MTT, Sigma) assays were performed. In brief, the pTi samples seeded with MC3T3-E1 cells were incubated with 80 μL MTT at 37 °C for 4 h. Then, 800 μL dimethyl sulfoxide (DMSO) was added to dissolve the formazan formed by MTT. The mixture was then transferred to 96 well plate and the optical density (OD) values were determined at 490 nm with a multimode microplate reader (Tecan GENios. San Jose, CA, USA).

### *In vitro* osteogenesis-related gene expressions in osteoblasts

Total RNA was isolated from the MC3T3-E1 cells attached to the implants using TRizol (Invitrogen, Carlsbad, CA, USA) according to the manufacturer’s protocol and quantified using a spectrophotometry (SmartSpec Plus, Bio-Rad, Hercules, CA, USA). 2 μg RNA was reverse-transcribed into cDNA in 40 μL system with oligo(dT)_18_ as a primer using FastQuant RT Kit (Tiangen Biotech, Beijing, China). RT-PCR was performed on 2 μL cDNA in a reaction of 20 μL system with Maxima SYBR Green qPCR (Thermo Fisher Scientific, Waltham, MA, USA) using the Bio-Rad CFX96 real-time PCR detection system (Philadelphia, PA, USA). The primer sequences utilized in semi-quantitative RT-PCR are shown in Table 1. The protocol for semi-quantitative RT-PCR reactions consisted of an initial denaturation at 95 °C for 10 min followed by 40 cycle denaturation at 95 °C for 15 sec, annealing at 55 °C for 15 sec, and extension at 55 °C for 15 sec. β-Actin was used as an internal control for normalization. The relative quantity of mRNA was calculated (2^−△△Ct^ analysis).

### *In vitro* osteogenesis-related protein expressions in osteoblasts

The MC3T3-E1 cells attached to the implants were washed with ice-cold PBS and lysed to release the whole proteins by RIPA buffer with 1 mM PMSF. The cell lysates were transferred into a pre-cooled microfuge tube and agitated for 30 min at 4 °C. The protein extracts were then centrifuged at 4 °C for 20 min. The protein content of the supernatant was collected and the protein concentration was determined by the BCA assay using a BCA protein assay kit (Pierce Chemical, Rockford, IL). The protein extracts (30 μg per sample) were subjected to electrophoretic separation by 10% Tris-glycine SDS-PAGE and transferred onto PVDF membranes (Millipore) after mixed with 2× loading buffer and boiled for 5 min. The PVDF membranes were blocked in TBST (Tris Buffer Saline, 0.5% Tween-20) containing 5% BSA for 1 h and incubated overnight at 4 °C with primary antibodies to Runx2 (1 × 10^−3^ mg/ml), Wnt1 (1 × 10^−3^ mg/ml), Lrp6 (0.5 × 10^−3^ mg/ml), β-catenin (0.2 × 10^−3^ mg/ml), β-Tubulin (1 × 10^−3^ mg/ml), and β-Actin (1 × 10^−3^ mg/ml) in TBST containing 5% BSA. All primary antibodies were purchased from Abcam (Cambridge, MA, USA). The membranes were then incubated with a 1:3000 dilution of HRP-conjugated goat anti-rabbit secondary antibody (Bioworld Technology, Minneapolis, MN, USA) for 1 h, and then visualized by an ECL chemiluminescence system (ImageQuant 350, GE Healthcare, Piscataway, NJ, USA). Semi-quantitative analysis was performed using the QuantityOne Software (Bio-Rad, Hercules, CA, USA). β-Tubulin or β-Actin was used as the internal control for normalization.

### Bone defect animal model

All procedures in the experiment were approved by the Institutional Animal Care and Use Committee of the Fourth Military Medical University, and all procedures were strictly carried out in accordance with the approved guidelines. Twenty-four female New Zealand rabbits with 14.5 ± 2.3 weeks of age weighting 2.9 ± 0.4 kg (Animal Center of the Fourth Military Medical University, Xi’an, China) were used in this study. Rabbits were acclimatized to the laboratory for 7 days before surgery. All animals were anesthetized via intramuscular injection with 3% pentobarbital sodium (30 mg/kg). Left hindlimbs of rabbits were shaved, cleansed with iodophor solution, and then covered with sterile drapes. A longitudinal incision in the distal femur was placed to expose the lateral condyle. A cylindrical bone defect with 6.0 mm diameter and 8.0 mm length were created with an electrical drill. The drill-hole defect was then washed with saline and hydrogen peroxide, and filled with a matching size cylindrical block of pTi. The incisions in the muscle, subcutaneous tissue and skin were then sutured, respectively. All surgical procedures were performed aseptically to avoid the potential infection of pathogens. At 1 day post surgery, all rabbits were examined using a digital radiography system (Carestream Health DRX-1, Rochester, NY, USA) under anaesthesia with pentobarbital sodium to further confirm the accuracy of the location of bone defect and orientation of pTi implants. Rabbits received intramuscular injection of penicillin (40000 U) for three consecutive days after surgery. One week post surgery, animals were then randomly and equally assigned to the Control and PEMF groups. Rabbits in the PEMF group were subjected to 2 h/day whole-body PEMF stimulation. All animals received intramuscular injections of 8 mg/kg calcein (Sigma) on 14 and 4 days before sacrifice to label mineralizing surfaces for dynamic bone histomorphometric analyses. After PEMF stimulation for 6 and 12 weeks, 6 rabbits in each group were euthanatized with an overdose of pentobarbital sodium. The femoral condylar samples were immediately harvested and immersed in 80% ethanol for μCT, histological and histomorphometric analyses. The femoral bone with 1 cm height right above the bone defect site was snap-frozen in liquid nitrogen for semi-quantitative RT-PCR analyses.

### μCT analysis

Left femoral condyles of rabbits in each group (*n* = 6 femora in each time point) were scanned using a high-resolution μCT system (Y. Cheetah, YXLON, Germany). After scanning, 2-D image sequences were transferred to a workstation and 3-D images were reconstructed with an 18.2 μm isotropic voxel size. A tube volume with 6.0 mm diameter and 8.0 mm length was defined as the volume of interest (VOI), which completely covered the region of the pTi implant. The trabecular bone parameters, including bone volume per total volume (BV/TV), bone surface per bone volume (BS/BV), trabecular number (Tb.N), trabecular thickness (Tb.Th) and trabecular separation (Tb.Sp) were quantified.

### Histology and histomorphometry

After μCT scanning, all bone samples were embedded in polymethyl methacrylate. Then, samples were sectioned longitudinally along the pTi implants (~50 μm thick) using the LEICA 2500E diamond saw microtome (Leica SpA, Milan, Italy). The sections were imaged using fluorescence microscope (LEICA DM LA, Leica Microsystems, Heidelberg, Germany) to quantify the dynamic histomorphometric parameters, including mineral apposition rate (MAR, the average distance between the two calcein labels divided by the labeling time intervals), mineralizing surface per bone surface (MS/BS, single-labeled surface plus one half of double-labeled surface in percentage of bone surface) and bone formation rate per bone surface (BFR/BS, calculated as MAR*MS/BS). After calcein double-labeling imaging, samples were subject to Masson-Goldner trichrome staining to further evaluate the cancellous bone histology. The parameter of bone area fraction was quantified from the pixels that represented bone tissue (bone area per total area) in the Masson-Goldner trichrome staining images.

### *In vivo* osteogenesis-related gene expressions

Before RNA extraction, samples were immediately crushed into powder in a mortar containing liquid nitrogen using the pestle and then mixed with the monophasic solution of phenol and guanidine thiocyanate. Total RNA was extracted using the guanidinium isothiocyanate-alcohol phenyl-chloroform method. Then, the FastQuant RT Kit was used to synthesize cDNA from RNA. RT-PCR was performed on the Bio-Rad CFX96 real-time PCR system. The primer sequences utilized in semi-quantitative RT-PCR are shown in Table 2. All mRNA levels were normalized by the house-keeping gene glyceraldehyde 3-phosphate dehydrogenase (GAPDH). The relative quantity of mRNA was calculated (2^−△△Ct^ analysis).

### Statistical analysis

All data presented in this study were expressed as the mean ± standard deviation (S.D.). Statistical analyses were performed using SPSS version 13.0 for Windows software (SPSS, Chicago, IL, USA). All data were examined for normal distribution using the Kolmogorov-Smirnov test. The homogeneity of variance was evaluated using the Levene’s test. Analyses showed that each specific parameter for the *in vitro* or *in vivo* data obeyed normal distribution and homoscedasticity. For all the *in vitro* experimental data and *in vivo* semi-quantitative RT-PCR results, the differences of each parameter between the Control group and PEMF group were examined using a Student t-test. For μCT, histological and histomorphometric analyses, one-way analysis of variance (ANOVA) was employed for evaluating the existence of differences among the four groups, and once a significant difference was observed, Bonferroni’s post hoc analysis was used to determine the significance between each two groups. *P* < 0.05 was considered statistically significant.

## Results

### *In vitro* osteoblast attachment, proliferation and morphology in pTi

**Figure 2:**
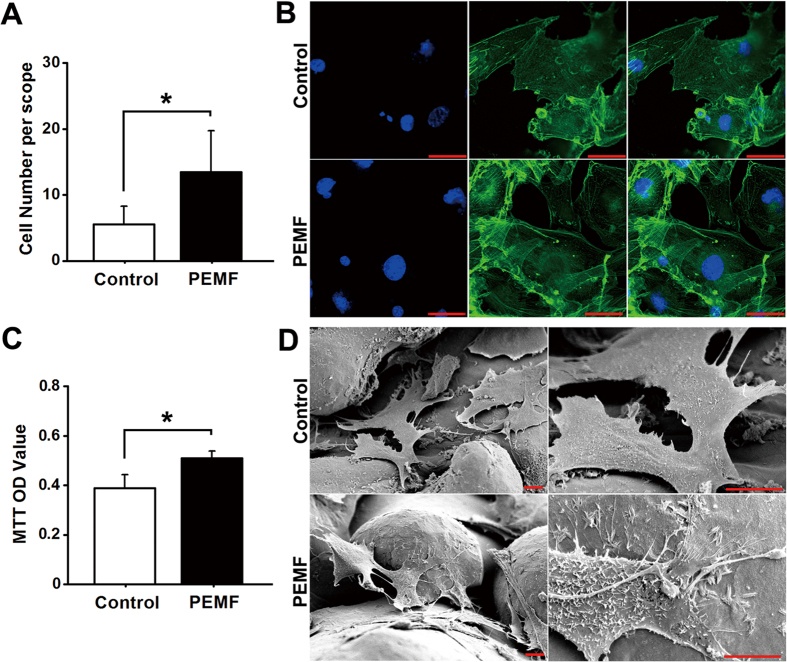
Effects of PEMF exposure on *in vitro* cellular attachment, proliferation and morphology for osteoblasts seeded in pTi. **(A)** Comparisons of *in vitro* osteoblast attachment between the Control and PEMF exposure groups via DAPI staining (*n* = 15). **(B)** Representative *in vitro* FITC cytoskeleton staining images of osteoblasts in the Control and PEMF exposure groups. Scale bar represents 50 μm for all images. **(C)** Comparisons of *in vitro* osteoblast proliferation between the Control and PEMF groups via MTT assays (*n* = 9). MTT was added into *in vitro* MC3T3-E1 cells to form the formazan, and DMSO was then added to dissolve the formazan. The optical density (OD) values of the mixture were determined at 490 nm with the multimode microplate reader. **(D)** Representative SEM scanning for *in vitro* osteoblasts in the Control and PEMF groups. Scale bar represents 10 μm for all images. Values are all expressed as mean ± S.D. *Significant difference from the Control group with *P* < 0.05.

As shown in Fig. 2A, PEMF exposure significantly increased cellular attachment for osteoblasts seeded in pTi as compared with the Control group via DAPI staining (*P* < 0.05). Representative *in vitro* FITC cytoskeleton staining images of osteoblasts (Fig. 2B) show that cells in the PEMF group displayed well-developed cytoskeleton with higher fluorescence intensity, more microfilaments and thicker stress fibers. In contrast, cells in the Control group showed lower cell number and poorly-organized cytoskeleton. Moreover, statistical comparisons further demonstrate that PEMF stimulation significantly promoted cellular proliferation for osteoblasts seeded in pTi via MTT analyses (Fig. 2C, *P* < 0.05). SEM scanning shows that cells were proliferated with more pseudopodia in pTi under PEMF stimulation (Fig. 2D). Images with higher magnification reveal that the PEMF-stimulated group shows more ruffled membranes, and more lamellipodia and filopodia as compared with the Control group.

### *In vitro* osteogenesis-related gene and protein expressions

**Figure 3:**
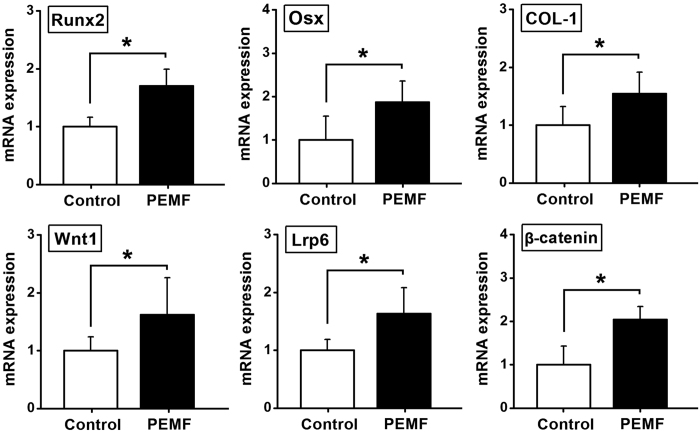
Effects of PEMF exposure on *in vitro* osteogenesis-related gene expressions for osteoblasts seeded in pTi via semi-quantitative RT-PCR analyses, including Runx2, Osx, COL-1, Wnt1, Lrp6 and β-catenin. Values are all expressed as mean ± S.D. (*n* = 8 ~ 11) and the relative expression level of each gene was normalized to β-Actin. *Significant difference from the Control group with *P* < 0.05.

**Figure 4:**
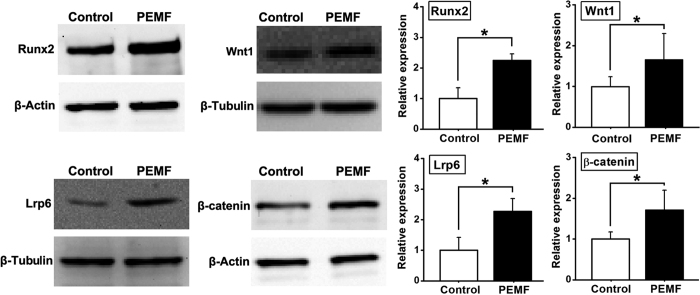
Effects of PEMF exposure on *in vitro* osteogenesis-related protein expressions for osteoblasts seeded in pTi via western blotting analyses, including Runx2, Wnt1, Lrp6 and β-catenin. Values are all expressed as mean ± S.D. (*n* = 3 ~ 4). The relative protein expression levels of Runx2 and β-catenin were normalized to β-Actin, and the relative protein expressions of Wnt1 and Lrp6 was normalized to β-Tubulin. *Significant difference from the Control group with *P* < 0.05.

The results of *in vitro* osteogenesis-related gene and protein expressions for osteoblasts seeded in pTi are shown in Figs 3 and 4. In comparison with the Control group, PEMF stimulation significantly promoted the expressions of osteogenesis-related genes via semi-quantitative RT-PCR analyses (Fig. 3), including Runx2, Osx and COL-1 (*P* < 0.05), and also increased the gene expressions of canonical Wnt signaling, including Wnt1, Lrp6 and β-catenin (*P* < 0.05). The results of western blotting analyses (Fig. 4) reveal that *in vitro* osteogenesis-related protein expressions for osteoblasts seeded in pTi, including Runx2, Wnt1, Lrp6 and β-catenin were significantly higher in the PEMF-stimulated group than those in the Control group (*P* < 0.05).

### μCT analysis for *in vivo* osseointegration of pTi

**Figure 5:**
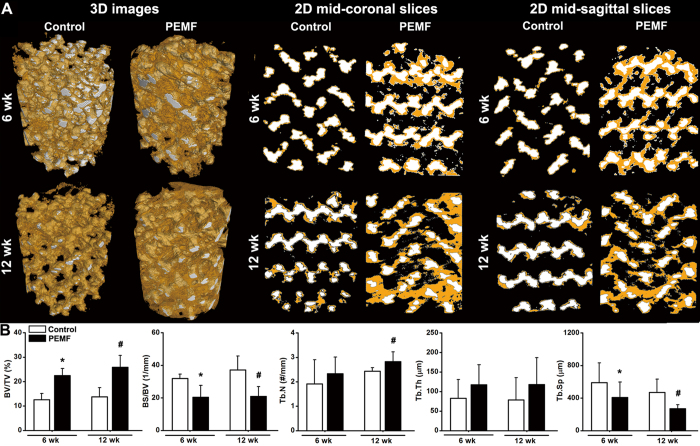
Effects of 6-week and 12-week PEMF exposure on the osseointegration of pTi implants in the region of bone defect via μCT scanning. A tube volume with 6.0 mm diameter and 8.0 mm length was defined as the volume of interest (VOI), which completely covered the region of the pTi implant. (**A**) Reconstructed 3-D μCT images determined by the VOI and 2-D mid-coronal and mid-sagittal slices. The regions with white color represent titanium alloys and the areas with yellow color represent cancellous bones. **(B)** Quantitative comparisons of μCT characteristic parameters of trabecular bones between the Control and PEMF groups (*n* = 6), including bone volume per tissue volume (BV/TV), bone surface per bone volume (BS/BV), trabecular number (Tb.N), trabecular thickness (Tb.Th) and trabecular separation (Tb.Sp). Values are all expressed as mean ± S.D. *Significant difference from the Control group at 6 weeks with *P* < 0.05. ^#^Significant difference from the Control group at 12 weeks with *P* < 0.05.

Representative 3-D and 2-D μCT images for *in vivo* osseointegration of pTi in the rabbit femora with bone defect are shown in Fig. 5A. As indicated by 3-D μCT images, PEMF exposure for 6 weeks and 12 weeks significantly increased the amount of newly formed bone within the implants as compared with the Control group. Images of 2-D mid-coronal and mid-sagittal slices further confirmed that bone ingrowth through the pores of pTi was significantly promoted by 6-week and 12-week PEMF stimulation. Quantitative statistical comparisons (Fig. 5B) demonstrate that PEMF exposure for 6 weeks and 12 weeks resulted in significant increase of BV/TV (*P* < 0.05, +78.5% at 6 weeks and +88.0% at 12 weeks). PEMF stimulation for 6 weeks and 12 weeks also significantly decreased the levels of BS/BV (*P* < 0.05, −36.2% and −43.6%) and Tb.Sp (*P* < 0.05, −30.7% and −42.8%). Moreover, PEMF exposure caused higher levels of Tb.N at 6 weeks (*P* > 0.05, +21.7%) and 12 weeks (*P* < 0.05, +16.3%), and also increased Tb.Th at 6 weeks (*P* > 0.05, +41.8%) and 12 weeks (*P* > 0.05, +49.6%). However, no significant difference was observed in BV/TV, BS/BV, Tb.N, Tb.Th and Tb.Sp between 6-week and 12-week PEMF exposure groups (*P* > 0.05).

### Histological and histomorphometric evaluation for *in vivo* osseointegration of pTi

**Figure 6:**
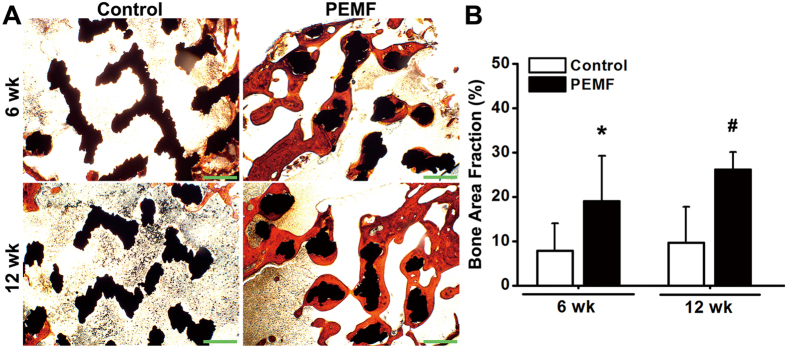
Effects of 6-week and 12-week PEMF exposure on cancellous bone histology in the region of bone defect via Masson-Goldner trichrome staining. (**A**) Representative histological images for bone microarchitecture in the region of bone defect by Masson-Goldner trichrome staining. The black areas represent titanium alloys and the red areas represent cancellous bones. Scale bar represents 100 μm for all images. (**B**) Quantitative comparisons of bone area fraction (bone area per total area) determined by the histological analyses between the Control and PEMF groups (*n* = 6). Values are all expressed as mean ± S.D. *Significant difference from the Control group at 6 weeks with *P* < 0.05. ^#^Significant difference from the Control group at 12 weeks with *P* < 0.05.

**Figure 7:**
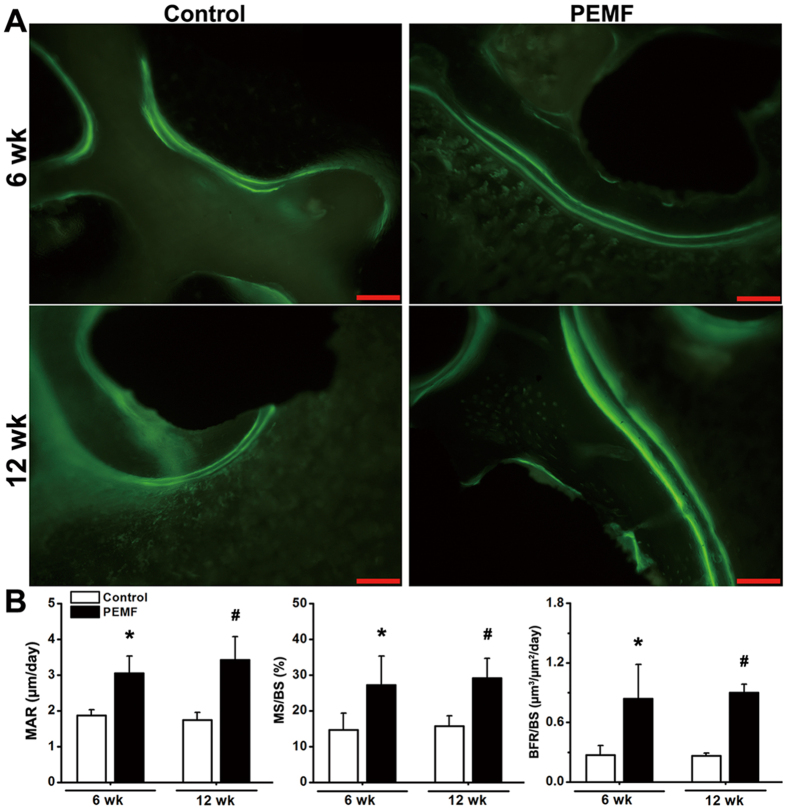
Effects of 6-week and 12-week PEMF exposure on dynamic histomorphometric parameters in the region of bone defect via calcein double-labeling analyses. (**A**) Representative calcein double-labeling sections in the region of bone defect. Scale bar represents 100 μm for all images. (**B**) Quantitative comparisons of the dynamic histomorphometric parameters, including mineral apposition rate (MAR), mineralizing surface per bone surface (MS/BS) and bone formation rate per bone surface (BFR/BS) between the Control and PEMF groups (*n* = 6). Values are all expressed as mean ± S.D. *Significant difference from the Control group at 6 weeks with *P* < 0.05. ^#^Significant difference from the Control group at 12 weeks with *P* < 0.05.

Representative histological images (Fig. 6A) by Masson-Goldner trichrome staining demonstrate that PEMF exposure stimulated more new trabecular bone ingrowth through the pores of pTi in the region of bone defect. Statistical comparisons of the histological analyses (Fig. 6B) further reveal that the levels of bone area fractions were significantly higher in the PEMF-stimulated group (*P* < 0.05, +143.1% at 6 weeks and +169.8% at 12 weeks). Dynamic histomorphometric analyses via calcein double-labeling staining (Fig. 7A) show that PEMF stimulation speeded up the new bone formation in the region of bone defect. Quantitative comparisons (Fig. 7B) reveal that 6-week and 12-week PEMF stimulation significantly increased MAR (*P* < 0.05, +62.7% and +96.3%), MS/BS (*P* < 0.05, +85.6% and +85.6%) and BFR/BS (*P* < 0.05, +209.3% and +239.9%) as compared with the Control group. However, no significant difference was found in bone area fraction, MAR, MS/BS and BFR/BS between 6-week and 12-week PEMF exposure groups (*P* > 0.05).

### *In vivo* osteogenesis-related gene expressions

**Figure 8:**
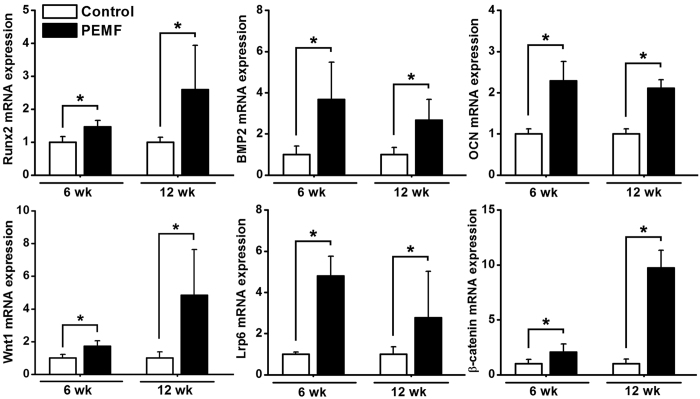
Effects of 6-week and 12-week PEMF exposure on *in vivo* osteogenesis-related gene expressions in rabbit femora via semi-quantitative RT-PCR analyses, including Runx2, BMP2, OCN, Wnt1, Lrp6 and β-catenin. Values are all expressed as mean ± S.D. (*n* = 6) and the relative expression level of each gene was normalized to GAPDH. *Significant difference from the Control group with *P* < 0.05.

The results of *in vivo* osteogenesis-related gene expressions via semi-quantitative RT-PCR analyses are shown in Fig. 8. PEMF exposure for 6 weeks and 12 weeks significantly promoted osteogenesis-related gene expressions as compared with the Control group, including Runx2, BMP2 and OCN (*P* < 0.05). Moreover, mRNA levels of the canonical Wnt signaling pathway (including Wnt1, Lrp6 and β-catenin) in the PEMF group were also significantly higher than those in the Control group (*P* < 0.05).

## Discussion

Titanium alloys especially at porous structures have shown great potential for the application in orthopedics. Exploring effective approaches for enhancing the repair efficiency and quality of titanium implants in bone defect remain critical and significant clinical topics. In the present study, PEMF exposure, as a kind of safe and non-contact biophysical intervention, was employed to determine its capacity for improving the osseointegrative properties of pTi implants. Our findings clearly demonstrate that PEMF stimulation significantly promoted osteoblast functions and activities in pTi *in vitro* and enhanced osteogenesis and bone ingrowth of pTi implants *in vivo* in bone defect animals. Moreover, we also reveal that canonical Wnt signaling might be involved in the promotion of osseointegration of pTi implants by PEMF stimulation. Our findings suggest that PEMF might become a potential biophysical modality for enhancing the repair efficiency and quality of pTi implants in bone defect.

The spatial magnetic action and electric currents are able to be generated surrounding the bone tissues when the skeletons are exposed to exogenous PEMF stimulation. Titanium implants, as typical nonferromagnetic materials, possess excellent properties of electrical conduction, but not disturb or shield the spatial magnetic flux distribution^34^,^35^. Thus, the favorable electromagnetic compatibility of titanium implants provides theoretical feasibility for the combined pTi and PEMF therapy for bone defect. In the present study, the structural parameters of pTi, including the porosity and pore size have been widely used in previous investigations both experimentally and clinically^7^,^36^,^37^. Our μCT and histological staining results reveal the limited bone ingrowth through the pores 7 weeks and 13 weeks post surgery, which necessitate the treatment for the enhancement of osseointegration of pTi. In the group receiving PEMF exposure, more new trabecular bone formation was observed at the implant-bone interface as well as in the center of the implants, as evidenced by the μCT and static histomorphometric results. Our findings revealed faster and higher-quality osseointegration under the PEMF exposure, which may lead to long-term stability and durability of implant fixation, as well as better overall mechanical performance of whole bone^38^,^39^. Furthermore, calcein double labeling-based histomorphometric analyses were used to evaluate the effects of PEMF on bone remodeling in pTi implants. Our dynamic bone histomorphometric results demonstrate the modulating role of PEMF in bone remodeling with obvious anabolic effects at the bone defect site. PEMF exposure significantly increased the bone formation rate in pTi implants, as revealed by increased MAR, MS/BS and BFR/BS. These results keep consistent with our previous findings for the regulatory role of PEMF in bone metabolism in osteoporotic rats^20^. However, we did not observe significant difference in μCT, histological and histomorphometric parameters between 6-week and 12-week PEMF exposure, indicating that 6-week PEMF stimulation may provide adequate osseointegration and bone ingrowth of pTi implants. Moreover, our PCR results further demonstrate that PEMF exposure significantly promoted *in vivo* gene expressions of Runx2, BMP2 and OCN, revealing obvious promotive effects of PEMF on osteoblastogenesis in peri-implant bones^40^. Taken together, our *in vivo* results show that PEMF stimulation has the capacity of enhancing bone ingrowth and osseointegration in pTi implants by promoting bone anabolism.

To further examine the promotional effects of PEMF exposure on the biocompatibility of pTi with bone cells, *in vitro* osteoblasts seeded into three-dimensional pTi discs were subjected to PEMF stimulation with the same parameters as the *in vivo* investigation. Our results demonstrate that PEMF stimulation enhanced the proliferation of osteoblasts *in vitro* in three-dimensional pTi during the cellular active proliferation stage via MTT and fluorescence staining observations, which keep consistent with previous studies on monolayer cells^24^,^41^ or cells seeded on two-dimensional flat titanium surface^27^. Our findings also reveal that PEMF stimulation facilitated the initial adhesion of osteoblasts to pTi. The potential mechanism might be associated with the polarization of cell membrane and increased protein adsorption to the pTi surface induced by the electrical potential generated by PEMF^27^,^42^. Besides the increased cell amount, osteoblasts in pTi also exhibited significantly distinct microstructure and cytoskeletal organization after PEMF simulation. Cells in the PEMF group exhibited more ruffled membranes and many more pseudopodia and microfilaments. These changes in cellular structures were considered to be essential events for bone cells detecting and transducing the external biophysical signals, and thus regulating the biological behavior of bone cells (*e.g.*, proliferation and differentiation)^43^,^44^,^45^. Moreover, our findings also showed significantly up-regulated expressions of biomarkers for osteoblast differentiation and mineralization (Runx2, Osx and COL-1) in PEMF-stimulated group^40^,^46^,^47^, revealing the potential stimulatory efficiency of PEMF on osteogenesis for osteoblasts *in vitro* seeded in pTi. Thus, our findings suggest that PEMF stimulation was able to promote the biological activities and functions of *in vitro* osteoblasts in three-dimensional pTi implants.

To unravel the mechanism by which PEMF exposure modulated osseointegration and bone remodeling in pTi implants, gene expressions of osteoblastogenesis-associated canonical Wnt signaling pathway were systematically investigated both *in vivo* and *in vitro*. Wnts, as a family of secreted proteins existing extensively within the skeleton, can bind to the cell membrane Frizzled and Lrp5/6 co-receptors, and consequently lead to the stabilization of β-catenin in the cytoplasm and promote more Wnt-targeted gene transcription^48^,^49^. Activation of canonical Wnt signaling can increase bone formation via multiple routes, including promoting the differentiation of mesenchymal stem cells into mature osteoblasts, enhancing the proliferation and mineralization of osteoblasts, and preventing the osteoblast apoptosis^49^. A large body of evidence has also revealed the importance of canonical Wnt signaling in regulating the expressions of osteogenesis-related cytokines^50^,^51^,^52^. Zhang and colleagues found that Wnt/β-catenin signaling was able to activate BMP2 gene expressions of osteoblasts^53^. Studies by Gaur *et al*. have also shown that canonical Wnt signaling promotes osteogenesis by directly stimulating Runx2 gene expression both *in vitro* and *in vivo*^54^. It has also been shown that Wnts have the capacity of up-regulating the expression of Osx^55^. In the present study, our semi-quantitative RT-PCR and western blotting results demonstrate significant increases of gene and protein expressions of canonical Wnt signaling for *in vitro* osteoblasts seeded in pTi in PEMF-stimulated group, including Wnt1, Lrp6 and β-catenin. Moreover, our findings also reveal that PEMF augmented the *in vivo* gene expressions of Wnt1, Lrp6 and β-catenin of peri-implant bones, revealing that canonical Wnt signaling has been implicated in regulating PEMF-induced improvement of osteogenesis in pTi implants in the repair of bone defect. These findings keep consistent with *in vivo* findings, revealing PEMF-induced activation of canonical Wnt signaling in osteoporotic rats^20^,^32^,^56^. Together, our results indicate that PEMF might regulate osteoblastogenesis and new bone formation in pTi implants through a potentially primary mechanism of promoting the activation of canonical Wnt signaling.

There are also some limitations in the present study. First, our *in vitro* experiment was performed on murine-derived MC3T3-E1 osteoblast cell line, which was different with the animal species used for our *in vivo* investigation. However, our findings reveal PEMF-induced enhancement on the biological activities of mouse MC3T3-E1 cells *in vitro* and bone ingrowth of pTi in rabbit bone defect *in vivo*, confirming the positive efficiency of PEMF on osteogenesis and osseointegration of pTi. Second, our present *in vivo* experimental technique was still unable to fully elucidate the cellular microenvironment mechanism for PEMF-induced acceleration of osteogenesis and osseointegration surrounding the implant. Osetoblasts, osteoclasts and macrophages as well as mesenchymal and hematopoietic stem cells may be all involved in this process of osseointegration. This issue will be systematically clarified in the following *in vivo* investigations. Third, we herein created the cylindrical bone defect in the femoral lateral condyle. The reason for selecting this region as the defect site is that it contains abundant trabecular bone which is much easier to investigate and quantify osteogenesis and bone ingrowth of pTi. However, it should be noted that most posttraumatic defects occur in the diaphysis. Thus, investigating the effects of PEMF on osseointegration of pTi in the diaphyseal cortical bone will be also an interesting and important aspect in the succeeding study.

## Conclusion

In conclusion, the present study represents the first report demonstrating that PEMF stimulation promoted bone ingrowth and osseointegration of pTi implants via obvious anabolic actions in the repair of bone defect. Our findings also reveal significantly beneficial effects of PEMF exposure on the biological activities and functions of *in vitro* osteoblasts seeded in pTi implants. Moreover, activation of Wnt1, Lrp6 and β-catenin was observed both *in vitro* and *in vivo* in PEMF-stimulated group, revealing the involvement of canonical Wnt signaling in promoting osteogenesis in pTi implants. Our findings suggest that pTi implants accompanied by PEMF exposure exhibit high efficiency and quality in the repair of bone defect, and might become a clinically applicable treatment modality for osseous defects.

## Additional Information

**How to cite this article**: Jing. *et al*. Pulsed electromagnetic fields promote osteogenesis and osseointegration of porous titanium implants in bone defect repair through a Wnt/β-catenin signaling-associated mechanism. *Sci. Rep.* **6**, 32045; doi: 10.1038/srep32045 (2016).

## Figures and Tables

**Table 1:** The sequence of primers used in the present study for *in vitro* real-time fluorescence quantitative PCR.

Genes	Primers	Primer Sequence (5′-3′)	Product Length (bp)
Runx-2	Forward	TGCACCTACCAGCCTCACCATAC	105
	Reverse	GACAGCGACTTCATTCGACTTCC	
Osx	Forward	TATGGCTCGTGGTACAAG	200
	Reverse	TCAGATGGGTAAGTAGGC	
COL-1	Forward	GAAGGCTGGAGAGCGAG	132
	Reverse	CGGGACCTTGTTCACCTC	
Wnt1	Forward	ATTTTGGTCGCCTCTTTG	140
	Reverse	GTGGCATTTGCACTCTTG	
Lrp6	Forward	CAGCACCACAGGCCACCAA	227
	Reverse	TCGAGACATTCCTGGAAGAG	
β-catenin	Forward	GGAAAGCAAGCTCATCATTCT	171
	Reverse	AGTGCCTGCATCCCACCA	
β-Actin	Forward	GCCAACACAGTGCTGTCT	114
	Reverse	AGGAGCAATGATCTTGATCTT	

**Table 2:** The sequence of primers used in the present study for real-time fluorescence quantitative PCR analysis in rabbit bones.

Genes	Primers	Primer Sequence (5′-3′)	Product Length (bp)
Runx-2	Forward	CAGTCTTACCCCTCTTACC	130
	Reverse	CATCTTTACCTGAAATGCG	
BMP2	Forward	GGACGACATCCTGAGCGAGT	117
	Reverse	CGGCGGTACAAGTCCAGCAT	
Osteocalcin	Forward	TTGGTGCACACCTAGCAGAC	216
	Reverse	ACCTTATTGCCCTCCTGCTT	
Wnt1	Forward	CTCCACGAACCTGCTAACTG	226
	Reverse	GACGATCTTGCCGAAGAGG	
Lrp6	Forward	GCTTGGCACTTGTATGTAAA	179
	Reverse	TGGGCTAAGATCATCAGACT	
β-catenin	Forward	GACACGGACCACACGCACAA	173
	Reverse	CCGAGCAGCAGCAAGTCTTCT	
GAPDH	Forward	CATCATCCCTGCCTCCACTG	183
	Reverse	GATGCCTGCTTCACCACCTT	
